# Effects of field parameters on IMRT plan quality for gynecological cancer: A case study

**DOI:** 10.1120/jacmp.v6i3.2087

**Published:** 2005-08-17

**Authors:** Albert Y.C. Fung, Charles A. Enke, Komanduri M. Ayyangar, Robert B. Thompson, Weining Zhen, Natarajan V. Raman, David Djajaputra, Sicong Li, Ramasamy M. Nehru, Sushakumari Pillai, Paul Sourivong, Mary Headley, Ann L. Yager

**Affiliations:** ^1^ Department of Radiation Oncology Nebraska Medical Center 987521 Nebraska Medical Center Omaha Nebraska 68198‐7521 U.S.A.

**Keywords:** gynecological, radiation, IMRT, treatment planning

## Abstract

Traditional external beam radiotherapy of gynecological cancer consists of a 3D, four‐field‐box technique. The radiation treatment area is a large region of normal tissue, with greater inhomogeneity over the treatment volume, which could benefit more with intensity‐modulated radiation therapy (IMRT). This is a case report of IMRT planning for a patient with endometrial cancer. The planning target volume (PTV) spanned the intrapelvic and periaortic lymph nodes to a 33‐cm length. Planning and treatment were accomplished using double isocenters. The IMRT plan was compared with a 3D plan, and the effects of field parameters were studied. Delineated anatomical contours included the intrapelvic nodes (PTV), bone marrow, small bowel, bladder, rectum, sigmoid colon, periaortic nodes (PTV), spinal cord, left kidney, right kidney, large bowel, liver, and tissue (excluding the PTVs). Comparisons were made between IMRT and 3D plans, 23‐MV and 6‐MV energies, zero and rotated collimator angles, different numbers of segments, and opposite gantry angle configurations. The plans were evaluated based on dose‐volume histograms (DVHs). Compared with the 3D plan, the IMRT plan had superior dose conformity and spared the bladder and sigmoid colon embedded in the intrapelvic nodes. The higher energy (23 MV) reduced the dose to most critical organs and delivered less integral dose. Zero collimator angles resulted in a better plan than “optimized” collimator angles, with lower dose to most of the normal structures. The number of segments did not have much effect on isodose distribution, but a reasonable number of segments was necessary to keep treatment time from being prohibitively long. Gantry angles, when evenly spaced, had no noticeable effect on the plan. The patient tolerated the treatment well, and the initial complete blood count was favorable. Our results indicated that large‐volume tumor sites may also benefit from precise conformal delivery of IMRT.

PACS numbers: 87.53.Kn, 87.53.Tf

## I. INTRODUCTION

It is estimated that there are 83 000 new cases of gynecological cancer in the United States in 2004 and 29 000 deaths per year.^(^ ^1^ ^)^ Half the gynecological cancer cases originate at the uterine corpus. The American Cancer Society estimated that “a malignant endometrial tumor will develop in approximately 700,000 of the 48 million women in the United States aged 35 or older.^(^ ^2^ ^)^ For late‐stage disease with metastasis to the intrapelvic and para‐aortic lymph nodes, treatment often involves surgery and postoperative radiation therapy and/or chemotherapy. The five‐year survival rate for lymph node positive disease ranges from 20% to 60%.^(^ ^2^ ^)^ The radiation therapy regimens typically consist of external beam radiation and/or intracavitary brachytherapy. Traditional external beam technique uses a 3D, four‐field box with shielding by multileaf collimators (MLCs).^(^ ^3^ ^)^ This four‐field‐box‐technique includes a substantial amount of normal tissue in the treated field, causing morbidity and complication.

Intensity‐modulated radiation therapy (IMRT) is one of the most significant technological advances in radiation therapy in the past decade. A majority of radiation clinics in the United States have implemented IMRT or plan to implement IMRT in the near future. The common treatment sites related to IMRT include head and neck, prostate, and intracranial tumors. Gynecological treatment has not been a common area for IMRT. A recent survey indicated that IMRT was being performed on 88% of head and neck patients, while only 15% of gynecological cancer radiation therapy patients were treated with IMRT.^(^ ^4^ ^)^ The conventional thinking is that IMRT is more suitable for small‐volume tumors, while late‐stage gynecological cancer, with a large volume of lymph nodes as target volume, is not an appropriate candidate for IMRT. While there is some truth to this, the opposite argument is equally valid. Radiation for gynecological malignancies delivers extensive dose to a large region of normal tissue, which may benefit from the technical capabilities of IMRT.

There are several reports in the literature on IMRT planning for gynecological malignancies. Mundt et al.^(^ ^5^ ^)^ compared conventional whole pelvic radiation therapy plans to IMRT plans for 10 patients (5 cervical, 5 endometrial). They found that IMRT resulted in more conformal dose and a reduction of irradiated volume in small bowel, rectum, and bladder. Heron et al.^(^ ^6^ ^)^ did a similar comparison on 10 gynecological patients and arrived at the same conclusion. Adli et al.^(^ ^7^ ^)^ investigated the IMRT planning of 16 patients and discovered that prone positioning with a belly board decreased the small bowel even more. The published articles on gynecological IMRT have used one single isocenter for all treatment beams.

This report is a case study of IMRT planning in a patient with endometrial cancer. The planning target volume (PTV) spanned the periaortic and the pelvic lymph nodes to a length of 33 cm. This was larger than the IMRT capability of MLCs in most of the LINACs. Planning and treatment were accomplished using two separate isocenters. All critical organs in the abdominal and pelvic regions were delineated, and their dose distributions were investigated. The IMRT plan was compared with a traditional 3D plan. The field parameters in IMRT planning were also studied: beam energy, collimator angles, the number of segments, and gantry angles, to observe the effect of these parameters on the final dose distribution.

## II. METHODS

The patient is a 53‐year‐old female who had FIGO^(^ ^8^ ^,^ ^9^ ^)^ stage IIIC, grade 3 endometrial adenocarcinoma. The patient had undergone total abdominal hysterectomy with bilateral salpingo‐oophorectomy and pelvic as well as periaortic lymphadenectomy and pelvic washings. One of the 8 lymph nodes from left pelvic node dissection and one of 12 from the right were positive for metastatic carcinoma. Four periaortic lymph nodes were recovered, all of which were negative for malignancy spread. It was decided to proceed with postoperative radiation therapy followed by systemic chemotherapy.

The patient was simulated for radiation treatment with CT. Simulation was done in a supine posture with the arms above the head. A Vac Lok® (MED‐TEC Inc., Orange City, IA) cradle was used for immobilization, and a styroform block was placed between the taped feet. CT contrast was not used. The Pinnacle radiation planning system (Philips Medical Systems, Bothell, WA) was used for the IMRT and 3D planning, and radiation was delivered with segmental MLCs of a Siemens Primus LINAC (Siemens Medical Solution, Erlangen, Germany). The goals of radiation therapy were to treat both the pelvic and periaortic lymph nodes to a dose prescription of 45 Gy in 1.8‐Gy fractions, while minimizing the risk for small bowel morbidity and sparing bone marrow. The bone marrow would be important for chemotherapy afterward. Thirteen anatomical contours in the pelvic and abdominal regions were delineated in the plan. The planning target volumes, as defined in ICRU Report 50,^(^ ^10^ ^)^ were intrapelvic and periaortic nodes. The tissue structure included all the tissue in the CT image series with the target volumes (intrapelvic and periaortic nodes) excluded. The dose to tissue would give a measure of the “integral dose” delivered.

We studied the effect on the plan quality by various treatment parameters: energy, collimator angle, number of segments, and gantry angles. Table 1 lists the parameters of the “original” plan (the original parameters were used for the actual treatment of the patient), as well as the alternative parameter settings for plan comparison in this study. The whole PTV, including intrapelvic and periaortic nodes, traversed 33 cm in the superior‐inferior direction and 15 cm in the lateral direction. The total target volume was 1293 cm^3^. Since the target volumes were lymph nodes, it was not possible to separately distinguish the gross (GTV) or clinical (CTV) target volume; hence, the physician directly drew the PTV. Although the Primus MLCs permitted a maximum field size of 40 cm at the isocenter, only the middle 27 cm of a 1‐cm leaf width could be used for IMRT delivery. Therefore, it was necessary to split the treatment field into two isocenters: one at the pelvis and the other at the abdomen, 15 cm from each other. The original IMRT plan had a total of 14 beams, that is, 7 beams centered at each isocenter, with evenly spaced gantry angles starting at 0° (anterior).

**Table 1:** Parameters of the “original” plan (the original parameters were used for the actual treatment of the patient), as well as the alternative parameter setting for plan comparison in this study

Test	Parameter to compare	Original plan	Compared plan
1	modality	IMRT	3D
2	energy of X‐ray	23 MV	6 MV
3	collimator angle	zero	rotated
4	number of segments	165	509
5	gantry angle	start at 0° (anterior)	start at 180° (posterior)

The dose “objectives” used in the optimization that generated the original IMRT plan are listed in Table 2. As stated, it was possible to put in more than one objective for each region of interest (ROI). The critical organs included in the optimization objectives were all in the pelvic region, except for the bone marrow, which extended from the abdomen to the pelvis. The same objectives were used in all the IMRT plans for fair comparison. The number of iterations used in optimization was 25 because the total cost function was observed to be stabilized (i.e., not decreasing any more) well before the 25th iteration. For quality assurance, a hybrid plan composed of the treatment beams irradiating the virtual water phantom was generated, and the doses at the two isocenters were verified with ion chamber measurement.

**Table 2:** Objectives used in the optimization that generated the original IMRT plan

Region of interest	Type	Target (cGy)	% Volume	Weight
intrapelvic nodes	uniform dose	4500		40
periaortic nodes	uniform dose	4500		15
bone marrow	max dose	3000		0.5
small bowel	max dose	3000		0.6
bladder	max dose	3000		0.5
rectum	max dose	3000		0.5
rectum	max DVH	3000	50	0.5
sigmoid colon	max dose	2000		0.5
sigmoid colon	max DVH	1500	50	0.5

Test 1 compared the IMRT plan with a 3D non‐IMRT plan. The typical 3D plans had fewer numbers of radiation beams than the IMRT plans. Our alternative 3D plan had 4×2=8 fields, giving 4‐field box distributions. The MLC apertures of the 3D portals were designed based on the beam's‐eye views (BEVs) of the PTVs. The collimator settings for the 3D plan were different than the IMRT plan, especially at the junction between the pelvic beams and the abdominal beams to ensure proper field matching.

**Figure 1:**
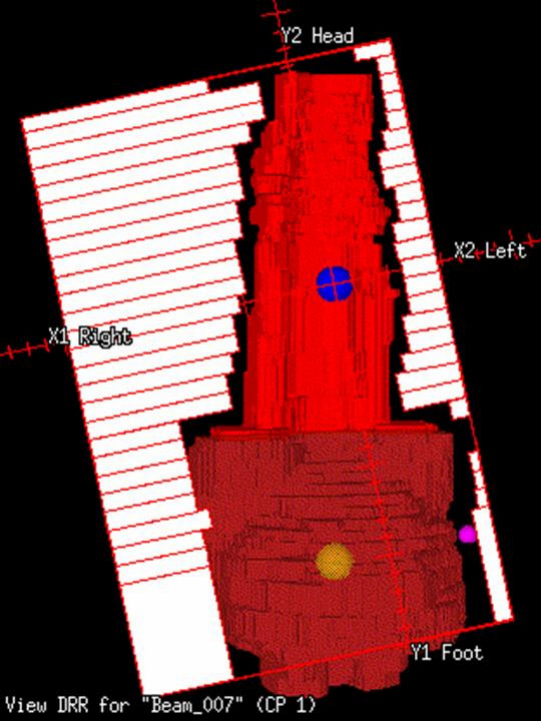
Beam's‐eye view (BEV) of field #7 at the abdominal isocenter in the collimator‐rotated plan. By rotating the collimator angle to make the PTV more “diagonal” in the BEV, the abdominal beams could encompass more of the pelvic PTV, and the pelvic beams could encompass more of the periaortic PTV.

In Test 2, the IMRT optimization was a rerun from iteration one with the same settings and objectives. The only change was the energy, which changed from 23 MV to 6 MV. Test 3 examined the effect of the collimator angle rotation. By rotating the collimator angle and making the PTVs more “diagonal” in the BEV, the abdominal beams could encompass more of the intrapelvic nodes (Fig. 1), and the pelvic beams could encompass more of the periaortic nodes. We studied whether this would result in a better plan. We used another planning system, the Nomos Corvus system (Sewickley, PA), to automatically determine the “optimal” collimator angle for each field relative to the shape of the PTVs. For each beam, the algorithm found the collimator angle so that the MLC leaves were perpendicular to the longest dimension of the combined PTVs. These rotated collimator angles were put in the ADAC Pinnacle system for IMRT optimization.

**Figure 2:**
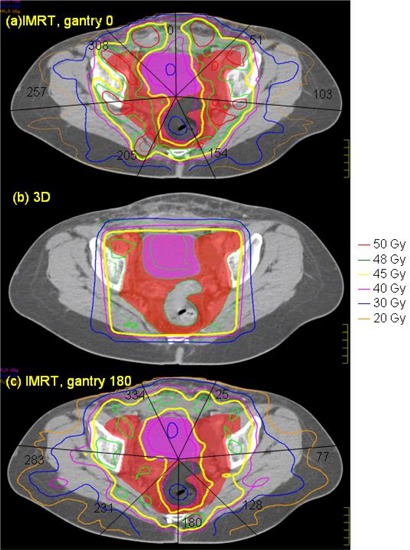
The 7 gantry angles overlaid on top of the pelvic isodose distribution for (a) the original IMRT, gantry 0 plan, (b) the 3D (gantry 0) plan, and (c) test 5 IMRT plan with opposite gantry angles. The bladder (purple) and the sigmoid colon (gray) are at the center and are deeply embedded in the intrapelvic nodes (red). Both IMRT plans tailored the prescription isodose (yellow) around the bladder and sigmoid colon, excluding these two organs from the highest dose. The 3D plan, as expected, delivered a convex uniform dose for the whole area, and the doses to bladder and sigmoid colon were as high as those of the PTVs.

Test 4 investigated the effect of the total number of MLC segments. The number of MLC segments determined the radiation‐on time, and we aimed at keeping it within 20 min. The original result from the Pinnacle optimization showed the intensity profile of each beam, which was then converted to MLC segments. Depending on the conversion criteria, the resulting MLC segments might give intensities slightly different than the ideal solutions, with fewer segments resulting in larger differences. All plans in the tests were reoptimized, resulting in slightly different segments. For Test 4, the alternative plan was converted to about three times the number of segments of the original plan. This would indicate how much improvement to expect from increasing the number of segments, although we were aware that the alternative plan would prohibitively take longer to deliver. Test 5 was a plan with all gantry angles inverted 180° and opposite to the original. The seven gantry angles are depicted in Fig. 2.

## III. RESULTS AND DISCUSSION

**Figure 3:**
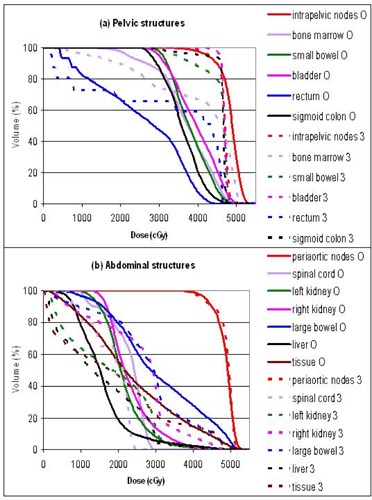
Test 1: DVH comparison between the original IMRT plan (O) and the 3D plan (3): (a) pelvic structures, (b) abdominal structures.

**Figure 4:**
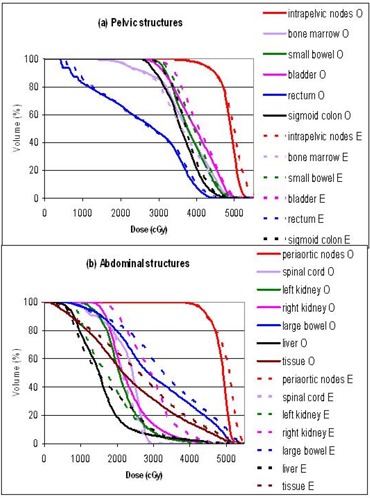
Test 2: DVH comparison between the original 23 MV plan (O) and the energy 6 MV plan (E): (a) pelvic structures, (b) abdominal structures.

**Figure 5:**
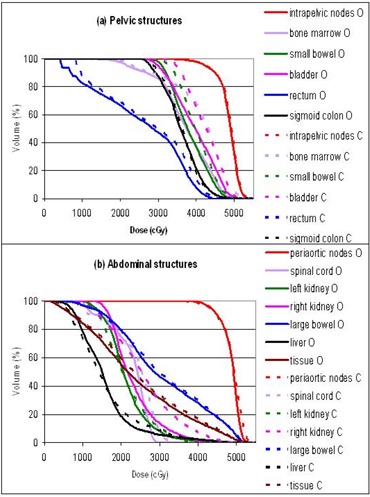
Test 3: DVH comparison between the original zero collimator plan (O) and the collimator‐rotated plan (C): (a) pelvic structures, (b) abdominal structures.

**Figure 6:**
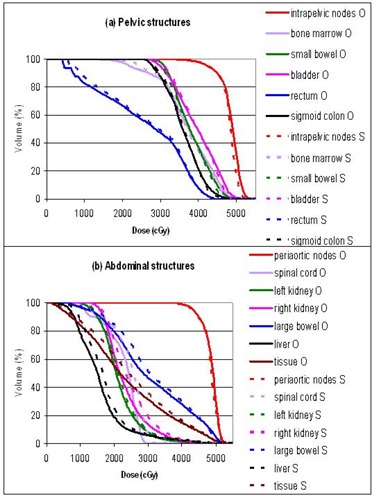
Test 4: DVH comparison between the original 165‐segment plan (O) and the 509‐segment plan (S): (a) pelvic structures, (b) abdominal structures.

**Figure 7:**
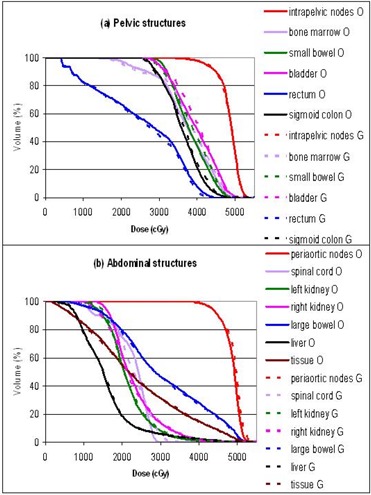
Test 5: DVH comparison between the original gantry‐start‐at‐0° plan (O) and the gantry‐opposite plan (G): (a) pelvic structures, (b) abdominal structures.

The plans were primarily evaluated by comparing the dose volume histograms (DVHs). Figures 3 to 7 show the DVHs of tests 1 to 5, respectively. For each test, (a) contained the structures in the pelvic regions, while (b) contained those in the abdominal regions. Bone marrow was included in the pelvic DVH graph, and tissue was included in the abdominal DVH graph arbitrarily. The symbols O, 3, E, C, and G stand for the original, 3D, energy 6 MV, collimator rotated, and gantry 180° plans, respectively. For the sake of this study, all the plans were normalized so that the DVHs of the intrapelvic and the periaortic nodes each had 90% of the volume receiving more than the prescribed dose of 45 Gy, that is, D90=45 Gy. Identical normalization ensured clinically relevant comparison. Table 3 summarizes the differences of each plan in every organ delineated. Table 4 lists the D10 (dose delivered to at least 10% of the volume) for every organ in each plan. D10 was chosen as a robust indicator of the maximum dose for each anatomical structure. The ranking in Table 3 might not agree with the numbers in Table 4, since Table 3 is based on the whole DVH curves, while Table 4 essentially represents single points on the DVH curves.

**Table 3:** Summary of the differences of each plan in every organ delineated

Anatomical structure	3D	Energy 6 MV	Collimator rotation	More MLC segments	Gantry opposite
intraplevic nodes	√	X	0	√	0
periaortic nodes	0	X	0	√	0
bone marrow	X	√	X	0	0
small bowel	XX	X	X	0	0
bladder	XX	X	X	0	0
rectum	XX	0	X	0	0
sigmoid colon	XX	X	0	0	0
spinal cord	√	√	0	X	0
left kidney	0	√	0	0	0
right kidney	X	0	X	X	0
large bowel	√	X	X	X	0
liver	X	X	0	X	0
tissue	√	X	0	X	0

√ = better than the original plan; 0 = similar to the original plan; x = worse than the original plan; xx = much worse than the original plan

**Table 4:** D10 (Gy) of each plan in every organ delineated

Anatomical structure	Original IMRT	3D	Energy 6 MV	Collimator rotation	More MLC segments	Gantry opposite
intraplevic nodes	51	48	53	51	50	51
periaortic nodes	51	51	51	51	51	51
bone marrow	46	48	46	48	45	46
small bowel	45	48	45	46	44	45
bladder	47	47	47	48	46	46
rectum	39	46	40	41	39	39
sigmoid colon	42	47	43	42	42	43
spinal cord	27	23	27	28	29	29
left kidney	29	30	29	29	30	29
right kidney	32	38	37	32	34	32
large bowel	48	45	50	48	48	48
liver	24	30	29	24	26	24
tissue	44	46	45	44	44	44

### A. 3D plan

The 3D plan delivered much higher dose to all the critical structures in the pelvic region, including the small bowel, bladder, rectum, and sigmoid colon. (Refer to the DVHs in Figs. 3 to 7 and the numbers in Table 4.) Figure 2 compares the isodose distributions at the pelvic level of (a) the original 23‐MV IMRT plan, (b) the 3D 23‐MV plan (test 1), and (c) the 6‐MV IMRT plan (test 2). The bladder (purple) and the sigmoid colon (gray) are at the center and are deeply embedded in the intrapelvic nodes (red). Both IMRT plans tailored the prescription isodose (yellow) around the bladder and sigmoid colon to exclude these two organs from the highest dose. The 3D plan, as expected, delivered a convex uniform dose for the whole area, and the doses to bladder and sigmoid colon were as high as that of the PTVs. Hence, the IMRT plan was more conformal than the 3D plan. It was found that the dose uniformity of the PTVs was better in the 3D than the IMRT plan. The dose to tissue, indicating the integral dose, was in fact less with the 3D plan. This represented a workable compromise to achieve IMRT conformity. The unexpected result here was that large bowel had lower dose in the 3D plan. IMRT, while keeping most of the critical organs in lower doses, had to deliver the radiation somewhere. In this case, higher dose was delivered to the large bowel with IMRT. Nevertheless, the 3D plan was overall considered inferior to the IMRT plan.

**Figure 8:**
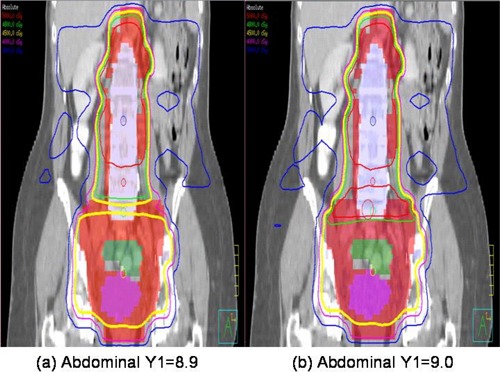
Coronal view of isodose distribution of two 3D plans with slightly different Y1s of the abdominal fields: (a) Y1=8.9 cm, (b) Y1=9.0 cm. When the field size was too small, a cold spot developed at the junction, while a one‐millimeter larger field size created a hot spot.

The 3D planning process has its own problem of field matching at the pelvic‐abdominal junction. Figure 8 shows the coronal view of isodose distribution of two 3D plans with slightly different Y1 jaw settings of the abdominal fields: (a) Y1=8.9 cm, and (b) Y1=9 cm. When the field size was too small, a cold spot developed at the junction, while a one‐millimeter increase in field size created a hot spot. The DVHs of the 3D plan in Fig. 3 were generated using Y1=8.9 cm. This demonstrates the importance of careful field matching in 3D planning. In contrast, no manual field matching was necessary for double isocenter IMRT. Dose nonuniformity at the junction was taken into account with the IMRT optimization objectives. The algorithm automatically generated a solution including field sizes and IMRT segments, and the resulting IMRT plans showed no problem at the pelvic‐abdominal junction. This is one of the key advantages of double isocenter IMRT.

### B. Energy 6 MV

Most anatomical structures received less desirable doses with 6 MV than 23 MV. The maximum doses in the intrapelvic and periaortic nodes were higher with 6 MV at the same normalization of D90=45 Gy, meaning worse PTV homogeneity with 6 MV. The doses to the small bowel, bladder, sigmoid colon, large bowel, and liver were also higher with 6 MV. Some critical organs, such as bone marrow, spinal cord, and left kidney, received lower doses with 6 MV. The integral dose in general, as indicated by the dose to tissue, was higher with 6 MV. Dose in the 6‐MV plan tended to spread out more into the surrounding normal structures, which seemingly made the 6‐MV plan inferior. However, recall that the plans only considered dose from photons and ignored the neutron dose. It is well known that high‐energy X‐rays (above 10 MV) generate neutrons from the LINAC, which may increase the possibility of secondary malignancy.^(^ ^11^ ^–^ ^13^ ^)^ In addition to the neutron dose to the patients with higher‐energy photon beams, there is another health risk by the neutron‐activated products^(^ ^14^ ^)^: the residual radiation after the treatment of higher‐energy IMRT can be considerable to therapists who enter the treatment room often. The physician should decide whether the photon dose advantage of higher energy is more significant than the neutron dose disadvantage.

### C. Collimator angle rotation

The rotated collimator angles as determined by the Corvus system are listed in Table 5. The plan with “optimized” collimator rotation was uniformly worse than the original plan with zero collimator angle. Bone marrow, small bowel, bladder, rectum, right kidney, and large bowel all received higher doses in the collimator rotated plan. There was not a single anatomical structure in which the collimator‐rotated plan fared better. We postulated that although the pelvic‐centered fields covered some of the periaortic nodes with rotated collimator, the 6.5‐cm wide superior end leaf of the MLC did not facilitate precise dose delivery, and the same for the inferior end leaf of the abdominal‐centered fields.

**Table 5:** Rotated collimator angles (in degrees) as determined by the Nomos Corvus system

	Gantry	205	257	308	0	51	103	154
pelvic	collimator	32	28	30	35	330	332	328
abdominal	collimator	350	29	345	9	16	24	12

### D. Number of segments

Optimization created different intensity profiles for each beam. A conversion from intensity profiles to MLC segments was necessary, and the converted MLC segments might not reproduce exactly the ideal intensity profiles. The Pinnacle conversion algorithm allowed criteria to discard segments that might not have a significant effect on total dose distribution. This kept the number of segments within a practical level. The original IMRT plan was generated with the following settings: error tolerance =7%, minimum segment area $=5\;{\text{cm}}^{2}$, and minimum segment monitor units (MUs)=4. That plan had a total of 165 segments for 14 fields. From the same optimization result, we also converted the MLC segments with error tolerance of 3%, no minimum segment area, and no minimum segment MUs. This gave a plan with 509 segments.

It was found that permitting more MLC segments resulted in better uniformity of the two PTVs. However, the plan with a higher number of segments delivered a higher dose to the abdominal critical organs, such as the spinal cord, right kidney, large bowel, and liver. It also resulted in a higher integral dose as indicated by the dose to normal tissue. A plan with more segments should be able to get better optimization results than one with fewer segments in general. The abdominal organ dose increased by 509 segments may be attributed to random occurrence. For this particular patient case, it was not possible to clearly conclude whether the plan with more or fewer segments was better. Given that a larger number of segments prolonged treatment time at the LINAC, an attempt to increase the number of segments in the hope of a better plan was determined impractical.

Incidentally, when the intensity profiles were different, the same conversion criteria did not guarantee exactly the same number of segments. Our test plans generated the following number of segments: original, 165; energy 6 MV, 164; collimator rotated, 150; segment test, 509; gantry 180°, 168.

### E. Gantry angle

The test plan used gantry angles complementary to the original plan. Hence, 0° became 180°, 205° became 25°, etc. It was generally believed that as long as the fields were evenly spaced around the treatment area, the exact gantry angles did not create a significant difference in achievable dose distribution. Our results confirmed this belief. The DVH curves of both gantry configurations were essentially similar for every anatomical structure. The two plans could be considered identical. The choice of gantry angles might be based on other considerations, such as couch interference during radiation delivery.

The above findings were based on evaluating each plan and all the DVHs as a whole. We were careful in our evaluation to not read too much into the specific DVH of each anatomical structure in this study (i.e., if bone marrow preservation is of top priority for a patient, among our plans the 6‐MV plan gave the best DVH, but that did not conclude that 6 MV was the method of choice in sparing bone marrow). Individual DVH results were probably due more to random chance and not a result of the absolute merit of that treatment parameter. The ranking of each structure in Table 3 was rather subjective, particularly on whether the pairs of DVHs were similar (0), so it would not be surprising if some readers disagree with a few of the rankings. Another limitation of our study was that the treatment plans did not explicitly take into consideration the motion of the various organs. The magnitude of patient movement in the abdomen and the pelvis likely had a significant effect on degrading the quality of radiation delivery.^(^ ^15^ ^)^ Tissue heterogeneity was ignored in all plans, that is, dose calculation was performed assuming unit density in all the tissue. Nevertheless, it was not expected that the inclusion of patient motion or tissue heterogeneity would alter the relative quality of the alternative plans. We chose not to investigate the effect of optimization objectives on isodose distribution, since any result might be peculiar to the Pinnacle planning system or the geometry of this particular patient.

Our results are in agreement with the findings from other investigators. Application of IMRT on a large abdominal target has been previously reported. Hong et al.^(^ ^16^ ^)^ studied 10 patients with whole‐abdomen radiation and discovered that IMRT resulted in significant dose reduction to the bones and improved PTV coverage as compared with conventional treatment. The high‐dose regions within the PTV increased slightly. Mundt et al.^(^ ^5^ ^)^ studied 10 patients with pelvic treatment and found that IMRT dose distribution (compared with 3D) was more conformal to the PTV, with better DVHs for small bowel, rectum, and bladder. They also found IMRT plans resulted in more dose inhomogeneity within the PTV. Heron et al.^(^ ^6^ ^)^ studied 10 patients treated with pelvic fields and concluded that the doses to small bowel, bladder, rectum were lower from IMRT versus 3D. Ahmed et al.^(^ ^17^ ^)^ studied 5 patients with abdominal treatment and demonstrated reduced doses to bone marrow, bowel, spinal cord, and both kidneys.

Our study was unique in several ways: the treated PTV (encompassing the intrapelvic and periaortic nodes with 33 cm total length) was among the largest of IMRT planning reported in the literature. This necessitated two isocenters and a total of 14 radiation fields. Our study was also meticulous in terms of delineating all critical structures involved in the abdominal and pelvic regions. Finally, while most investigations in IMRT gynecological treatment focused on comparison with the 3D plans, we also studied the effects of various IMRT parameters on isodose distribution. The discussion in literature of large‐volume IMRT treatment often included the limitation in field width of some models of LINAC (maximum 14.5 cm), and the consequent splitting of IMRT fields into twice the number of smaller subfields. The Primus LINAC had a limitation in field width of 20 cm, and we did not need to split any of our IMRT fields into smaller fields.

Since IMRT was developed in the past decade, most of the treatment delivered has been for small‐volume tumor sites, such as head and neck, prostate, and intracranial locations. There was an unspoken assumption within the radiation oncology community that large‐volume tumor sites were not suitable candidates for IMRT. We believe that this may be misplaced dogma and that large treatment fields encompassing extensive normal tissue may also benefit from precise conformal delivery of IMRT. IMRT allows for higher than conventional dose delivered to the target volume, while sparing critical organs from radiation toxicity. The patient tolerated this high‐dose treatment with remarkable condition, and the initial lab finding on complete blood count was favorable. It is expected that more research effort will be directed to this area, with adequate patient numbers and follow‐up to evaluate the clinical outcome of IMRT in gynecological treatment.

## IV. CONCLUSIONS

In summary, we have planned and treated a patient with endometrial carcinoma using IMRT. The PTV including the intrapelvic and periaortic lymph nodes had a length of 33 cm, and treatment was accomplished with two isocenters. In this case study, we compared the effect of various field parameters on the resultant plan. The findings were the following: the IMRT plan delivers a smaller amount of dose to critical organs compared with the 3D plan. High energies (23 MV) produce a more desirable plan because of lower total photon integral dose (ignoring neutron dose). Collimator rotation is unnecessary and, in fact, leads to a slightly inferior plan. Including more MLC segments does not improve plan quality significantly and certainly increases the treatment time. Switching the gantry angles to opposite directions does not change the isodose distribution in any meaningful way.

## ACKNOWLEDGMENTS

The authors would like to thank Philip M. Bruch, M.S., for constructive advice in preparing the manuscript.
